# Hermaphrodite, Gonochorist, and More: Navigating the Complexities of Sex-Related Language in Biology

**DOI:** 10.1093/icb/icag126

**Published:** 2026-07-20

**Authors:** A M Aramati Casper

**Affiliations:** Biology Department and Graduate Degree Program in Ecology, Colorado State University, Fort Collins, CO 80545, USA

## Abstract

In biology, the term *sex* is applied to numerous and entangled concepts, leading to language inconsistencies across taxa. Navigating this entanglement and complexity is vital because language not only influences how we communicate about a topic, it also directly shapes the way we conceptualize the world. Sex-related topics (e.g., sex; gender; sexual behavior; reproduction; and sexual, romantic, and related orientations) are areas where the language to describe these nuanced multidimensional spectra is challenging, because these topics are both complex and politically charged, even if we are focused on only communicating within biology. While there are ongoing discussions in biology to address current language needs, there is also the need to use language now, in both research and teaching. Teaching the complexity of sex-related topics in biology is essential, yet these topics are often oversimplified and overgeneralized in teaching contexts. To navigate the need for simplifications in teaching and other contexts, I call for the use of contextualized simplified content, in which one discusses biological complexity before focusing on simplifications. In this way, learners have a larger context for the simplifications they are being taught. The goal of this paper is to provide a resource that supports better usage of currently existing language in biology, allowing time for further development of discussions within biology that address these challenges. To do this, I synthesize terminology in the technical literature related to these topics, identify places where terminological usage has become muddy through oversimplification and overgeneralization, and identify spaces where more conceptual work in biology needs to occur to clarify meaning. While this text still involves some oversimplification, it represents an attempt to balance complexity and clarity in ways that are broadly accessible while inviting future conversations that are necessary to further create clarity in how we conceptualize and communicate about the topics of sex, gender, sexual behavior, and reproduction in biology.

## Introduction

Biology is inherently complex, full of fuzzy edges that can be difficult to conceptualize and describe. Having, using, and, as needed, developing language that can communicate nuance is important because language influences how we are able to think and communicate and shapes the way we conceptualize the world (Winawer et al. 2007; O’Connor 2019). Therefore, language can both limit and facilitate teaching and research in biology. While these challenges are relevant broadly in biology, this paper focuses on topics related to sex (sex; gender; reproduction; sexual behavior; and sexual, romantic, and related orientations), which are important for understanding biology from organism-to-ecosystem scales and across developmental, evolutionary, and ecological perspectives.

There are numerous concepts that the term *sex* is applied to in biology (e.g., McLaughlin et al. 2023; Massa 2026; McDonough-Goldstein et al. 2026; Warkentin et al. 2026a). And, within this range of concepts, there is a breadth of entangled language that is inconsistent across taxa and often problematically oversimplified (Cardoso et al. 2018; Casper et al. 2022; Zemenick et al. 2022; Pannell 2023). Although universal definitions may not be inherently desirable or feasible, clarity of meaning within a given context is important. Most simply, this can be achieved through a combination of word choice, explicitly defining the terms being used, and contextualizing simplifications when they occur. The goal of this manuscript is to (a) discuss the need for more clarity and specificity in the language we use for sex-related concepts in biology teaching and research and (b) increase the accessibility of these concepts and terms through serving as a resource that sits between oversimplified introductory textbooks and technical disciplinary texts.

### Importance of language: catalyst and constraint

Using accurate nuanced language in both research and teaching is critically important because the terms we learn and use directly shape the way we conceptualize the world (O’Connor 2019). For example, in some languages it is possible to reference a direction in relation to the speaker or an object (e.g., the tree is in front of the person), whereas in others directionality is always absolute in relation to the cardinal directions (e.g., the tree is to the north of the person). People who speak languages with absolute directions have better spatial awareness and navigational abilities, as they always must know where north is (O’Connor 2019). Therefore, a seemingly simple difference in language mechanics influences the way speakers move through the world.

Just like how relative or absolute directional language influences how one navigates spatially, in biology language constrains and catalyzes how we conceptualize life. And, because we move through the world as humans, we have particular ways of engaging with the world based on our lived experience, which we bring to the practice of science. When biology is taught focusing on oversimplified human-centric perspectives aligned with culturally dominant Euro-American world-views, such as cisheteronormativity, and fails to engage with concepts and language that describe biological diversity, biology actively creates harm through supporting artificially constrained perspectives rather than catalyzing expansive understanding of the true complexity of biology; conversely, teaching accurate biological variation and complexity helps counter harmful essentialist narratives (e.g., Donovan, Semmens, et al. 2019; Donovan et al. 2021, 2024; Casper et al. 2022; Blake et al. 2026).

Because of the human-centric bias that we all bring to everything we do, think about the assumptions you bring with you as you read this paper. Human-centric assumptions and anthropomorphization of other organisms can impede how people understand biology (Casper and Warkentin 2026; Nielson et al. 2025). Some words in this paper, such as *sex* and *gender* have multiple meanings. However, the lack of discrete boundaries for definitions or categories of different sex-related concepts does not mean that definitions and categories are not useful or that they do not exist. Instead, there are different definitions and categories useful in different contexts. In teaching, it is particularly important to discuss this contextual relevance of terms to help students understand which contextually specific meaning is relevant and to help them understand that the categories we develop to describe biological diversity are both constructed and defined by humans (Klopfer and Aikenhead 2022).

### Useful simplifications, or harmful oversimplifications?

Because of their conceptual and terminological complexity, sex-related concepts are often oversimplified from their broad multi-dimensional hyperspaces (Warkentin et al. 2026a) into discrete categories to make them easier to understand, particularly in teaching and everyday contexts. While some simplifications may be necessary and useful, the way sex-related concepts are commonly oversimplified can support essentialist thinking (Bazzul and Sykes 2011; Donovan et al. 2019; Donovan et al. 2024), where one expects members of a category to be relatively uniform in the characteristics used for classification (Coley and Tanner 2015). At best, these simplifications can create confusion and miscommunication among biologists when they interpret and use the same language to describe different concepts, particularly if they are working across sub-disciplinary boundaries (Warkentin et al. 2026a). More harmfully, these oversimplifications can uphold essentialists views, such as sexism and racism (Donovan et al. 2019; Donovan et al. 2020, 2021; Casper et al. 2022).

Analyses of common biology textbooks used in the secondary school through upper-level undergraduate level in both Canada and the US have demonstrated that these books commonly include oversimplifications about sex-related concepts that are in contrast to current biological knowledge (Bazzul and Sykes 2011; Fuselier et al. 2018; Ray King et al. 2021; Spaulding and Fuselier 2023; Donovan et al. 2024; Jackson et al. 2024). These oversimplifications include equating sex and gender, as well as presenting information as if there is little variation in behaviors or traits within a sex or gender group with little or no overlap between sex or gender groups, and with genes controlling the differences between sex or gender groups (Donovan et al. 2024). Essentialist beliefs cause direct harms. For example, fewer women obtain undergraduate and graduate degrees in STEM fields where gender essentialist beliefs are more common (Donovan et al. 2019), and queer students, who are underrepresented in STEM fields, experience a range of harms from gender and sex-related essentialism in biology curricula (Hughes 2018; Hughes and Kothari 2021; Casper et al. 2022; Maloy et al. 2022; Schneider et al. 2025).

Beyond the classroom, by supporting essentialist thinking, these oversimplifications allow for the misappropriation of biology to inaccurately support discriminatory social norms and legislation. This misappropriation is widespread, occurring in the US, the United Kingdom, and elsewhere, including in a report presented to the United Nations by a Special Rapporteurs appointee from Jordan (e.g., Miller 2024; The Florida Legislature 2024; Cochrane 2025; Langrand 2025; Massa 2026). Part of the broad harm of these laws is demonstrated by how, within the US, these laws are being used to limit federally funded research, and therefore the discourse that is even possible (Exec. Order No. 14168 2025). These harmful uses of oversimplified and incomplete biological knowledge demonstrate the vital need to engage with the complexity of these topics, including in how we use language in biology.

**Fig. 1:**
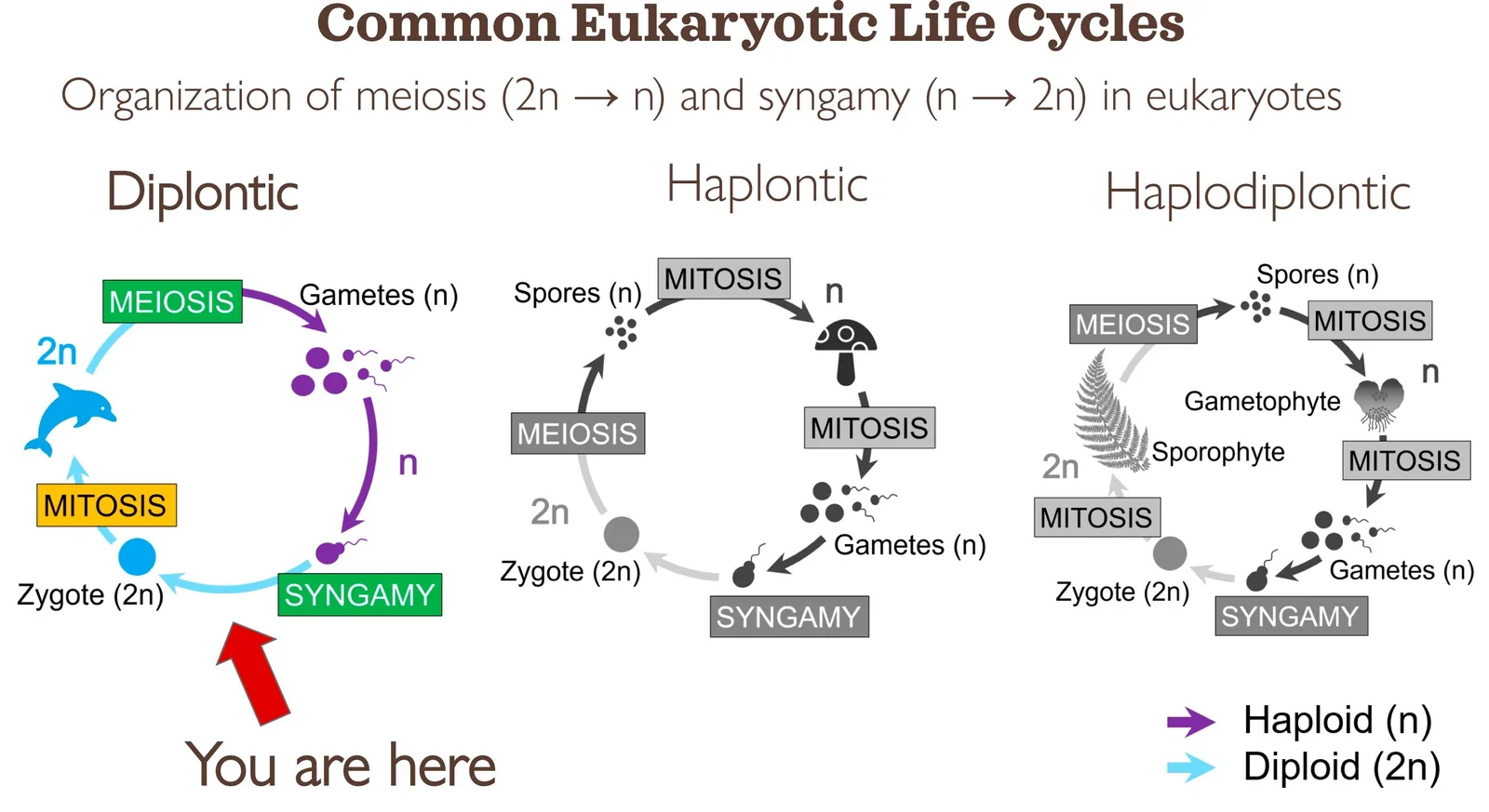
A course slide naming and contextualizing the diplontic life cycle. The fern leaf is in the public domain; the mushroom is by difatama and the dolphin is by Moreno, both from the Noun Project (CC-BY); and the fern gametophyte is by K.M. Warkentin.

Because of the aforementioned harms, engaging with complexity is important across biological contexts—normalizing complexity, rather than normalizing simplicity—sets up one’s expectations in research, teaching, and in broader every-day interactions. It conveys that life is full of variation and diversity. When simplifications are needed, contextualizing them within complexity provides a conceptual framework that shows how the simplifications relate to the broader complexity of life—a practice I’ve termed *contextualized simplified content*. In contextualized simplified content one forefronts variation, giving context for simplifications, and also discusses oversimplifications students may already be familiar with. For instance, when teaching about life cycles, one can start with naming the breadth of life cycles that exist, briefly comparing haplontic, diplontic, and haplodiplontic lifecycles, and normalizing that students are likely most familiar with our own life cycle, but may not have heard the name for it (see Eddy et al. 2026b for teaching materials, including Figures). Then, even if an instructor only discusses diplontic life cycles for the rest of the course, students have knowledge of both the name for our own diplontic life cycle, and that it is only one of multiple common life cycles (Fig. 1).

I developed the concept of contextualized simplified content from the intersection of calls to teach biological complexity (e.g., Long et al. 2021; Zemenick et al. 2022; Casper et al. 2025), challenges that students have in learning content when simply taught the biological complexity (Casper et al. 2026; Casper and Dockendorf 2023), and the pedagogical practice of cognitive bridging—helping to connect new material to students’ existing knowledge (Vidak et al. 2019).

### Challenges of sex-related language

One challenge in discussing and teaching about sex-related topics, which can lead to oversimplifications, is the complexity of and inconsistencies within sex-related language. Work to address inconsistencies in language is ongoing, such as through articulating the different concepts the term *sex* is used to describe, dismantling false binaries and articulating their complex variation, and identifying inconsistencies in how language is applied to different types of organisms (e.g., Fausto-Sterling 2012; Gorelick et al. 2016; Holleley et al. 2016; Sanz 2017; Maney et al. 2020; Heesch et al. 2021; Meissner 2021; Morgenroth and Ryan 2021; Diggle 2023; Goymann et al. 2023; McLaughlin et al. 2023; Oberle and Fairchild 2023; Pannell 2023; Subramaniam and Bartlett 2023; Eppley et al. 2026; McDonough-Goldstein et al. 2026; Warkentin et al. 2026a). Amidst this ongoing work, the goal of this paper is to provide a resource that supports the better use of currently existing language in biology by providing an accessible background to some of the complexity of sex-related topics across taxa, which can be used to contextualize simplifications one may need, such as when teaching.

This manuscript grew from the need, identified while writing Driessen et al. (2024) and in other conversations, for a resource that spans the gap between oversimplified introductory texts and dense disciplinary ones. In Driessen et al. (2024) we analyzed interviews with undergraduate-level biology instructors in the US to understand their approach to teaching about sex-related topics. We found that a major challenge instructors experienced in teaching complexity, rather than oversimplifications, was the specialized language involved, a finding that also parallels findings in some of my other research (Casper, Choi, et al. 2025). In one example in Driessen et al. (2024) this challenge occurred when an instructor was informed by a student that one should use the term *intersex* instead of *hermaphrodite*, and neither the student nor the instructor knew that the terms were not simply interchangeable. This current manuscript started as an appendix to the Driessen et al. (2024) manuscript to provide a resource for biology instructors about the complexity of sex-related topics, but rapidly grew beyond the bounds of an appendix.

### Journey through the diversity of sex

The vast biological diversity of sex-related topics would be hard to understand even if the terms were straightforward, which they are not. The goals of the following portions of the paper are to (a) explain key parts of the conceptual and terminological complexity of sex that is relevant in many biology courses, yet left out or oversimplified in introductory textbooks; (b) offer recommendations for better use of language with technically clear meanings for cases where uses have been muddied by common overgeneralizations and oversimplifications by nonspecialists (e.g., monoecious); and (c) identify areas where more conceptual work in biology is needed to clarify meaning (e.g., cosexual). Instructors may find some of the text boxes particularly useful, either directly as supplementary readings, or as references while creating their own materials.

While a paper of this length inevitably involves some simplification, I attempt to balance complexity and clarity in ways that are accessible for teaching, informed by research to identify instructor needs and develop and test teaching practices (Casper and Warkentin 2026; Casper et al. 2026; Eddy et al. 2026b; Long et al. 2021; Štrkalj and Pather 2021; Casper and Dockendorf 2023; Wright and Delgado 2023; Driessen et al. 2024, 2026; Casper et al. 2025; Blake et al. 2026). To bound the scope of my discussion, I focus on the subset of anisogamous species that produce eggs and sperm and primarily reproduce via meiosis and syngamy. However, the diversity of organisms beyond this subset, including isogamous organisms, non-oogamous anisogamous organisms, and anisogamous organisms that reproduce using parthenogenesis, apomixis, without gametes, or in the fuzzy boundaries between these categories, are vital to larger discussions around the diversity of sex, gender, and reproduction (Wiese et al. 1979; Wiese 1981; Xu 2004; Heitman et al. 2007 2007; Billiard et al. 2011; Booth et al. 2014; Cardoso et al. 2018; Constable and Kokko 2018, 2021; Wallen and Perlin 2018; Kratochvíl et al. 2020; Heesch et al. 2021; Lehtonen et al. 2021; Ryder et al. 2021; Laubscher et al. 2022).

### What is sex?

The term *sex* is used to characterize both organismal characteristics and processes. To parse out the different uses of the term, Warkentin et al. (2026a) present a framework of seven sex concepts and discuss each extensively. These are horizontal gene transfer **(sex0**), meiosis and syngamy (**sex1**), anisogamy (**sex2**), an organism’s anisogamete production strategy (e.g., only big gametes, only small gametes, or both types of gametes; **sex3**), the traits associated with anisogamete type/s produced (e.g., gamete-producing structures, hormone levels, chromosomes; **sex4**), syngamy-facilitating actions and interactions (**sex5**), and nonreproductive expressions and evolutionary derivatives of syngamy-facilitating actions (**sex6**). Casper and Warkentin (2026) present a convenient table with short descriptions of each concept. In Warkentin et al. (2026a), our goal is to facilitate conceptual clarity, not to identify one concept or use as “correct.” Here, I primarily focus on sex3 and sex4 because of their relevance for describing sex as a characteristic of an individual organism, not because they are the “correct” uses of *sex*.

Within this framework, the sex of an individual may vary across sex concepts and contexts: for example, chromosomal sex (a component of sex4) may be relevant in studying inheritance, but is not always aligned with the gamete type(s) an individual produces (sex3), including in humans and frogs, and is complicated in species such as the mice with X, X*, and Y chromosomes and the lizards that have gene-by-environment interactions that influence sex determination (Michala and Creighton 2010; Holleley et al. 2016; Lambert et al. 2019; Piferrer 2021; Saunders et al. 2022).

Organisms must be anisogamous (sex2) to have anisogamete production strategies (sex3) and associated characteristics (sex4). The variation in sex3 and sex4 characteristics is, in part, related to the fact that anisogamy (sex2) has evolved independently in many (over ten) distantly related eukaryotic lineages, with additional gains and losses within some clades (Henshaw et al. 2023; Warkentin et al. 2026a). Highlighting ways that sex2–4 vary among origins, noting the phylogenetic limits of homologous sex and breadth of analogous sex, may help instructors and researchers be more attuned to variation and avoid erroneous assumptions of similarity on the basis of assumed, overextended perceptions of homology (e.g., Oberle and Fairchild 2023).

### Overview of sexual systems and life cycles

Sexes, as organismal characteristics (i.e., sex3 and sex4), are influenced by and frequently considered as part of life cycles that involve both the exchange of genetic material through meiosis and syngamy (sex1) and the creation of new individuals (i.e., reproduction). Therefore, discussions of these sex concepts require an understanding of the life cycles they are embedded within. Yet, the centering of our own life cycle as an often unnamed norm situates all other life cycles as other, which can make learning about organisms with different life cycles more challenging (Casper and Warkentin 2026; Eddy et al. 2026b). Across three of the common eukaryotic life cycles (haplodiplontic, diplontic, and haplontic; see Text Box 1 for further descriptions; see Fusco and Minelli (2019) for additional life cycle complexity) there are a wide range of gonochoric and hermaphroditic sexual systems. Humans are **gonochoric**, where individuals typically produce either eggs or sperm during their life (Beukeboom and Perrin 2014; Leonard 2018). In contrast, individuals of **hermaphroditic** species typically produce both eggs and sperm at some point in their lifetime (Leonard 2018). Organisms within hermaphroditic species exist on a spectrum: some may be technically hermaphroditic, but primarily produce only one type of gamete, whereas others may produce closer to an even split of gametes. Both gonochoric and hermaphroditic species vary greatly at developmental and evolutionary levels regarding sex determination processes (e.g., genetic, environmental, and interactions between the two), morphology, and gamete production (Beukeboom and Perrin 2014; Leonard 2018), and all species can have individuals that produce no gametes during their life span.

Text Box 1: Common eukaryotic life cycles

Three common sexual life cycles in multicellular eukaryotic organisms are diplontic, haplontic, and haplodiplontic (Beukeboom and Perrin 2014; Fusco and Minelli 2019). They each describe different ways organisms move through processes of meiosis and syngamy, allowing for the recombination of genetic material while maintaining the number of copies of each chromosome across generations.

Each of these life cycle types is commonly described as moving between **haploid** (n, one full set of chromosomes) and **diploid** (2n, two full sets of chromosomes; see Eddy et al. 2026b for diagrams of each type of lifecycle). However, these life cycles can also involve moving between any multiple of n and 2n in polyploid organisms, which occur in animals, plants, fungi, and protists (Tomaszewska 2021). In all of these life cycles a diploid cell generally undergoes meiosis to become haploid, and two haploid cells undergo the processes of **plasmogamy** (the fusion of the cytoplasm of two cells) and **karyogamy** (the fusion of two haploid nuclei). While plasmogamy and karyogamy usually happen sequentially within a short time frame, one can happen without the other, or there may be a large time gap between the two (Banuett 2015). Some types of **parthenogenesis** (the growth of an individual gamete into a new individual) also involve variations of meiosis, karyogamy, and plasmogamy, and therefore are sometimes classified as sexual reproduction (Casper and Warkentin 2026; Beukeboom and Perrin 2014).

In many organisms the cells that undergo karyogamy and plasmogamy are gametes. While gamete is another term with fuzzy edges, **gametes** are haploid (or n) reproductive cells that typically fuse with another compatible cell to create a diploid (or 2n) zygote. However, gametes may grow in to a new organism on their own in parthenogenesis, and, depending on the parthenogenetic pathway offspring may be n, 2n, or polyploid (Richards 2003; Beukeboom and Perrin 2014; Heesch et al. 2021; Blanc et al. 2025).

Many animals, including humans, have **diplontic life cycles**, in which only the diploid life stage is multicellular. The haploid life stage exists as unicellular gametes, which, once produced through meiosis, then combine with another gamete, creating a diploid organism.

Many fungi have **haplontic life cycles**, in which only the haploid life stage is multicellular. However, it is also common for fungi to have more complex life cycles, including having dikaryotic cells, in which the cells have two different haploid nuclei (Banuett 2015).

All plants and some macroalgae have **haplodiplontic life cycles** (also referred to as **diplohaplontic**), in which both the haploid and diploid life stages are multicellular (Fusco and Minelli 2019; Heesch et al. 2021). In these life cycles, through the process of meiosis the diploid **sporophyte** (the organism that produces spores) produces haploid spores. **Spores**, in contrast with gametes, are reproductive cells that do not typically recombine with another reproductive cell to grow into a new organism. These spores grow into a haploid **gametophyte** (the organism that produces gametes), which then produces gametes, which are also haploid, through the process of mitosis. The gametes then combine via syngamy and grow into a diploid sporophyte.

Developmental variation in gonochoric species is often categorized into female, intersex, or male. While these classifications are based on biological characteristics, the category boundaries are socially constructed in an attempt to create discrete categories from a continuum (Fausto-Sterling 2012, 2019; Beischel et al. 2023; Massa et al. 2023). Furthermore, the terms *female, intersex*, and *male* can mean different things, and can be used to characterize an individual, such as a tree, or a part of an individual, such as a flower in a hermaphroditic plant. When thinking developmentally, one usually considers an organism’s sex4, the traits associated with the type of anisogamete produced, rather than actually measuring sex3, the gamete type an individual produces. In this case, **female** describes individuals that have the organ systems and other traits associated with making big gametes, often eggs, **intersex** describes individuals that have a mix of characteristics considered both typically female and typically male, and **male** generally characterizes individuals that have organ systems and other traits associated with making small gametes, often sperm (Leonard 2018). However, these sex4 traits do not inherently predict an individual’s sex3, the gametes they produce. Intersex individuals usually produce either one type of gamete or no gametes, but in some species they may produce both eggs and sperm; it is hypothesized that it is physically impossible for an individual in some gonochoristic species, including humans, to produce both eggs and sperm due to how their developmental pathways evolved (Sant’Anna et al. 2010; Bahamonde et al. 2013; Adolfi et al. 2019)^1^. However, this is only one way of using sex3 and sex4 characteristics to define the sex of an individual. Definitions using sex3, the gametes an individual produces, are often emphasized in biology because they are often useful in population and species-level contexts such as evolution, population biology, and ecology, but that use does not make sex3 inherently more correct than other definitions of sex.

In hermaphroditic species there are many ways that organisms can produce both sperm and eggs at some point in their lifetime (Beukeboom and Perrin 2014; Leonard 2018). Some hermaphrodites are sequential, others are simultaneous. Some can self-fertilize (some even obligately so), whereas others have a range of mechanisms that prevent self-fertilization (Costa et al. 2010; Cardoso et al. 2018). Some hermaphroditic species may have individuals that are all hermaphroditic, whereas others may include a mixture of hermaphrodites and males and/or females (Fusco and Minelli 2019). In the case of a hermaphroditic plant with flowers that are typically only male or female, the plant can also produce intersex flowers. Even though this nesting can occur, the terms intersex and hermaphrodite are not interchangeable.

### Considerations about relevance to human biology

Within this discussion of sexual systems and sex, it is necessary to consider that any discussions of humans involves describing the students learning in biology classes and the biologists doing research (Text Box 2). As is discussed in Text Box 2, some terms, like hermaphrodite, have been used to discriminate against intersex and queer people, and it is important to consider historical and current social contexts while using these terms. Therefore, even if the organisms being discussed are not closely related to humans, at least some students may interpret this information as relevant to their own and others’ lived experiences and biology (Casper et al. 2022; Driessen et al. 2024; Schneider et al. 2025; Eddy et al. 2026a). Language choice that affirms the variation of students in the course, including intersex and queer students, and centers diversity and variation as normal is important to support the wide range of learners in any biology course or space (for more about creating supportive classroom spaces, see Štrkalj and Pather 2021; Zemenick et al. 2022; Wright and Delgado 2023; Casper et al. 2025; Adams et al. 2026; Blake et al. 2026; Eddy et al. 2026a; Eddy et al. 2026b).

Text Box 2: Considering humans

Specific considerations are critical when discussing humans because we can communicate about our identities and be harmed by discriminatory language use. Some words and concepts have been applied to humans by people, including scientists, in exclusionary, derogatory, and discriminatory ways (Lerch and Servedio 2020; Viloria and Nieto 2020; Lewis and Sharpe 2023).

The term *hermaphrodite* is inappropriate to use for humans since humans, as a species, are gonochoric (Leonard 2018) and developmentally cannot produce both eggs and sperm (Adolfi et al. 2019). In biology, the term *intersex* is commonly used to describe characteristics of humans and other gonochoric organisms with sex3 and sex4 characteristics that do not clearly align with categories described as male and female. However, terminology for humans varies culturally (Costello 2016; Viloria and Nieto 2020). In the US clinical recommendations and some indeterminately sexed people use the term *people with DSDs* instead of *intersex*, for either *disorders of sex development* or *diversity of sex development* (Michala and Creighton 2010; Costello 2016).

For sex5 and sex6, biology is sometimes invoked to claim that heterosexual behavior is the only “biologically appropriate” behavior and evolutionarily all individuals should strive to reproduce, even though the concept of heterosexuality was only developed in western culture in the 19th century (Blank 2012). While behaviors existing in biology do not inherently make them right or wrong, understanding humans in a larger evolutionary context is still useful (Zemenick et al. 2022, 2023). Same-sex sexual behavior (sex6) occurs in more than 1,500 species of animals, is situated by current theory as evolutionarily beneficial, and is potentially an ancestral norm for animals (Bagemihl 1999; Bailey and Zuk 2009; Monk et al. 2019; Lerch and Servedio 2020; Gómez et al. 2023; Lerch 2026). Moreover, nonreproducing individuals are evolutionarily important in cooperative breeding species, such as humans (Hrdy 2011).

### Organization of sexes in bodies

Many characteristics used to define an individual’s sex3 and sex4 (and to define an individual) are based on **unitary body plans**, in which an individual consists of a single, genetically uniform and physiologically coherent unit (see Text Box 3 for a discussion of unitary and modular body plans; Vuorisalo and Tuomi 1986; Brewer and Smith 2011). However, characterizing sexes in ways that work for both unitary and modular organisms is ecologically, evolutionary, and developmentally relevant (Casper and Warkentin 2026; Cox 1988; Pannell 2023). A key way to start doing this is to simply discuss the unitary or modular nature of a given organism, and discuss differences between unitary and modular organisms in biology courses, rather than situating unitary organisms as normative (Casper and Warkentin 2026). While naming the unitary nature of an organism that has only one level of sexual organization may seem unnecessary, as unitary organisms are familiar to us, as humans, leaving them unnamed situates them as a false “normal,” in which modular organisms are an oddity (Casper and Warkentin 2026).

Text Box 3: Unitary and modular body plans

Eukaryotic organisms often have one of two types of body plans: modular or unitary. In addition, some species have complex life cycles in which they are modular in one life stage and unitary in another, and in others the boundary between modular and unitaryk is fuzzy (Fusco and Minelli 2019). The term ***modular organism*** was first developed to describe plants and defined using plant-specific language (Harper and White 1974; White 1979; Barthélémy and Caraglio 2007). However, the concept of modularity was expanded in the 1980s to include colonial animals (e.g., corals, bryozoans, and ascidians; Harper 1980; Hughes and Jackson 1985) and Vuorisalo and Tuomi (1986) proposed a definition for modularity that could encompass colonial animals: “**modules** are partially self-maintaining, repetitive, and multicellular parts of structural individuals” (p. 384). In contrast, unitary organisms, such as many animals including humans, can be thought of as being comprised of a single module, or a single unit that is a self-maintaining, multicellular, structural individual (Vuorisalo and Tuomi 1986).

The complexity of modular body plans means that there is a need for language that describes the organization of gamete-producing structures and gametes at each layer of modularity, such as the cone and tree level for a pine, as the number of organizational levels can range from two to at least five (Casper and Warkentin 2026; Cox 1988). While further work to describe the sex3 organization of modular organisms is needed, as a step towards filling this need Casper and Warkentin 2026 developed a diagramming system that allows for the characterization and comparison of unitary and modular organisms with different levels.

### Organization of gonochorists

The language describing gamete organization (i.e., sex3) in gonochorists is relatively simple, because even in modular organisms all levels are gonochoric. Language, such as dioecious/dioicous specifies this sameness (and also provides information about when sex3 is determined in the haplodiplontic life cycle) in modular organisms (see Text Box 4 for an explanation of the terms dioecious, dioicous, and other language the describes multiple layers of sexual modes in modular organisms.)

Text Box 4: Simultaneously describing layers of sexual modes in modular organisms

The terms dioecious, synecious, and monoecious provide information about reproductive structure organization for organisms with diploid-phase sex determination (e.g., vascular plants), with the parallel terms dioicous, monoicous, and synoicous for organisms with haploid-phase sex determination (e.g., nonvascular plants; Jackson 1928; Maciel-Silva and Porto 2014; Coelho et al. 2018). This difference exists because in vascular plants the sporophyte life stage is central in sex determination, because the sporophyte creates **megaspores** (large spores that grow into gametophytes that produce only macrogametes, often eggs) or **microspores** (small spores that grow into gametophytes that produce only microgametes, often sperm), whereas in bryophytes and some algae the haploid gametophyte life stage is central, because the sporophyte only creates one morphotype of spore and the sex2 of the gametes is determined by the gametophyte (Maciel-Silva and Porto 2014; Coelho et al. 2018; Cossard et al. 2022). Despite this difference, the set of vascular plant terms (dioecious and related) are also often used as general terms for all plants. While a broader term that captures both types of sex determination would be useful, this overgeneralized use of terms can lead to confusion and muddy the complexity they characterize (Warkentin et al. 2026b).

**Dioecious/dioicous** organisms only produce one type of gamete across their levels of organization, thus they have two types of individuals, those that produce eggs and those that produce sperm. Dioecious means “two houses,” one house for one type of gamete and another house for the other type of gamete (Rousseau, 1782, as cited in Jabbour et al. 2022). In angiosperms, flowers are **unisexual or imperfect**.

**Synecious/synoicous** organisms have one type of individual, those that produce both eggs and sperm in the same structure, such as a flower or a cone (Nath and Asthana 2001). In angiosperms synecious individuals produce **bisexual, hermaphroditic, or perfect flowers** (terms are synonymous; Jackson 1928). In these plants hermaphrodite may be used to describe both the reproductive structure (e.g., cone, flower) and the entire organism.

**Monoecious/monoicous** species also have one type of individual but have two types of reproductive structures on each individual, those that only produce eggs and those that only produce sperm. In angiosperms, flowers are **unisexual or imperfect** like in dioecious plants, but the entire organism is hermaphroditic. Monoecious means “one house,” for both type of gametes; the two sexes are in the same house (individual plant), but they do not share the same bedroom (e.g., flower in angiosperms; Rousseau, 1782, as cited in Jabbour et al. 2022; Sandford 2023). If they share the same “bedroom” they are synecious (if discussing only angiosperms monoclinous can also be used; Jackson 1928; Sandford 2023).

Even the above terms do not capture all the complexity in plants. For example, **trimonoecious** species have one type of individual that produces three kinds of reproductive structures: egg-producing and sperm-producing unisexual structures plus bisexual structures (Jackson 1928). And, there are many different arrangements of reproductive structures, such as flowers, that are characterized by other terms; these definitions only scratch the surface.

A further complexity in describing sexual organization in plant species is that it requires one to delineate between species. Yet, *species*, like *sex*, is a term that characterizes multiple concepts. Mayr (1940) concept of biological species was developed focusing on birds, and works poorly for plants and many other organisms (Hey et al. 2003; Haveman 2013).

### Organization in hermaphrodites

There are many ways to be a hermaphrodite. At a unitary level, hermaphroditic species exist in many ways that span simultaneous to sequential (Ghiselin 1974; Leonard 2018), and modular organisms can vary in these different “unitary” ways across their modules (Casper and Warkentin 2026; Wasson and Newberry 1997). **Simultaneous hermaphrodites** make both big and little gametes at the same time and **sequential hermaphrodites** switch the type of gametes they produce. Simultaneous hermaphrodites can also vary the proportions of the types of gametes they produce, from primarily producing one type of gamete and a few of the other type, to producing the same amount of each gamete type. Sequential hermaphrodites can switch in many ways (see Text Box 5). Some switch only once in their lifetime, whereas others switch daily (Groh et al. 2025; Falk 2026). While in some contexts it is useful to consider all hermaphrodites together, contrasting them with gonochorists, for many evolutionary and developmental questions it is crucial to distinguish sequential and simultaneous hermaphrodites. For instance, avoiding self-fertilization is easy for sequential hermaphrodites, but assuring fertility under partner scarcity requires special mechanisms (e.g., sperm storage), while simultaneous hermaphrodites face the opposite situation. Hence, some evolutionary analyses group sequential hermaphrodites with gonochorists, contrasting both with simultaneous hermaphrodites (e.g., Sasson and Ryan 2017). Developmentally, sex change requires different mechanisms than does maintaining concurrent production of eggs and sperm.

Text Box 5: Some of the many different ways of being a sequential hermaphrodite

Sequential hermaphrodites organize how they produce different types of gametes in time: they can be **protandrous**, sperm is produced before eggs; **protogynous**, eggs are produced before sperm; or **serial bi-directional**, when gamete type produced switches multiple times (Leonard 2018). Adolescent protogyny/andry and adolescent hermaphroditism describe organisms that switch from functionally gonochoric to hermaphroditic as they develop, or vice versa (Ghiselin 1974). When switching gamete production type, a sequential hermaphrodite may or may not produce both types of gametes for a time (Leonard 2018). Thus, sequential hermaphrodites that produce both eggs and sperm at the same time or that store sperm may also be able to “self-pollinate” or self-fertilize and may be subject to the strategies that limit these processes, as described for simultaneous hermaphrodites.

While protogynous (female then male) and protandrous (male then female) are often used to describe any organism that changes sex over time, they sometimes may be limited in use to individuals that do not produce both eggs and sperm during the same reproductive season (Davies and Singhal 1988). In this case, **cosexual** or sequential cosexual may be used to describe individuals that produce eggs and sperm during the same reproductive season (difference between cosexual and sequential cosexual unclear in text; Davies and Singhal 1988; Lang and Shain 2009). Differentiating the timing of gamete production can be important because each strategy may have evolved as a response to different selective pressures, which are also different from those that have led to simultaneous hermaphroditism (Ghiselin 1974; Davies and Singhal 1988). Furthermore, even in Ghiselin’s (1974) foundational text about the evolution of sex, there is no discussion of which terms should be used for organisms that reproduce year-round. This omission limits the applicability of the terms and may also represent a taxonomic bias (Culumber et al. 2019).

Relevant to simultaneous hermaphrodites, as well as sequential hermaphrodites that store sperm or have a brief overlap of gamete type production, it is common to use the terms *self-pollinating* and *self-fertilizing* to describe hermaphrodites that produce offspring through combining their own gametes. This differs from parthenogenesis because self-fertilization involves typical meiosis and syngamy (sex1) pathways. While many organisms can indeed self-fertilize, the term *self-pollination* oversimplifies the haplodiplontic life cycle of pollen-producing plants, because they are all **heterosporous (**sporophytes make two different types of spores, macrospores and microspores), with individual gametophytes only making one type of gamete (eggs or sperm). Therefore, ***self-pollination*** is actually the combination of gametes from two different individual gametophytes, which are half or full siblings produced by the same sporophyte (Meissner 2021; Sandford 2023). With that noted, angiosperms with bisexual flowers with **complete cleistogamy** are obligate “self-pollinators,” where the flowers never open, such as in some orchids; beyond obligate “self-pollinators,” in some angiosperms and gymnosperms the gametes produced by gametophytes with the same sporophyte parent are compatible with each other, as well as gametes with different sporophyte predecessor (Baskin and Baskin 2014; Cardoso et al. 2018). However some angiosperms have mechanisms that prevent successful “self-pollination,” which helps prevent offspring with the same sporophyte grandparent (Moore and Pannell 2011; Broz and Bedinger 2021). Strategies that prevent self-pollination, some of which parallel strategies that prevent self-fertilization, include genetic self-incompatibility, spatial organization of reproductive organs, and asynchronous maturation of eggs and sperm (Bedinger et al. 2017; Cardoso et al. 2018).

### Ongoing conceptual work

While the above-discussed terms and concepts have reached a general stability within many contexts in biology, there is still much ongoing conceptual work regarding different concepts of sex, even for just sex3 and sex4. Here, I discuss three examples of ongoing discussions about language and concepts within biology: the application of group-level categories to variable individuals, the use of the term *quantitative gender* in plants to characterize their sex3, and considerations for changing language in biology.

### Applying group-level categories to variable individuals

The language just discussed about gonochorists and hermaphrodites has been developed to discuss organisms generally, such as to characterize typical individuals within a species. Challenges arise at the individual developmental level when terms and concepts developed for and that work at a general level are used to strictly classify every individual (noting that there is no clear agreement on how to define an individual across taxa; Vuorisalo and Tuomi 1986; Wasson and Newberry 1997; Gorelick 2012). One example of how allowing for flexibility in classification, rather than using rigid pre-determined classifications, is beneficial is demonstrated by “leaky” sexual systems in plants. Because of the developmental variation in individuals in their study species, Anderson et al. (2021) characterized the complex reproductive system of *Plocama pendula* by using their floral morphology to label the flowers as strong hermaphrodites, weak hermaphrodites, or females. While this language is not what is universally used for *P. pendula*, the language allowed Anderson et al. (2021) to differentiate between floral morphologies in ways that were meaningful for answering the biological questions they were asking. Furthermore, they clearly define each sex category within the paper, and their terms simultaneously rely on the existing established definition of hermaphrodite while adding adjectives to differentiate between the different types of hermaphrodites. In this way they demonstrate an effective use of accessible and flexible language.

This example is only one of many that demonstrates how the relationship between sex3 and sex4 at an individual developmental level is highly variable and can be classified in a range of ways. Someone studying *P. pendula* with different research questions might classify the flowers differently; this demonstrates how categories in science are simultaneously rooted in observations and socially constructed. If one approached the study of *P. pendula* through forcing it into pre-defined categories that reflected assumptions not related to the biological variation present, it would limit researchers’ ability to understand the biology of these plants in many ways, ranging from reproductive success to ecological roles.

### Characterizing sex3 and reproductive success in plants: quantitative gender

Unlike the use of gender in humans (Massa 2026; McDonough-Goldstein et al. 2026), **quantitative gender** was a term first published by Lloyd in 1980 to provide information about sex in plants: it either describes either the proportion of eggs and sperm a plant makes (**phenotypic gender**; sex3), or the proportional contribution of a plant’s gametes to the next generation (**functional gender**; related to the success of sex5 facilitating sex2 and the growth of the resultant zygote; Lloyd 1979, 1980). Both are calculated with equations and are continua ranging from 0 to 1 (Lloyd 1980). Thus, an individual who makes equal amounts of eggs and sperm at a given time has a phenotypic gender of 0.5. Because phenotypic and functional genders describe labile characteristics, they represent “context-dependent and quantitative (nonbinary)” conceptualizations (Pannell 2023).

Phenotypic and functional gender are conceptually useful in plants, and, as we discuss in Casper and Warkentin (2026), these concepts have the potential to be useful more broadly in hermaphroditic organisms and in comparing hermaphroditic and gonochoric organisms. However, there is an ongoing discussion regarding if the term *gender* (and *male* and *female*) should continue to be used to describe plants. Oberle and Fairchild (2023) argue against this use because it creates confusion and isolates plant studies from the more general use of gender as a human social construct and sex as a biological concept. (See Beischel et al (2023), Fausto-Sterling (2019), and van Anders (2015), for a discussion of how many who study humans eschew this social/biological divide, argue for using the term gender/sex in humans, and describe how sex is a social construct.)

Others argue that accurately describing plant characteristics related to gamete and spore production requires moving away from not only gender but also *male* and *female* and developing new, more accurate language. Subramaniam and Bartlett’s (2023) suggestions include pistillate for ovule-producing flowers, staminate for pollen-producing flowers, and binate for flowers with both fertile stamens and pistils. However, the suggestions are angiosperm-centric, depending on specific reproductive structure anatomy. Meissner (2021) argues for a three and four sex model: sporophyte, male, and female for plants, such as ferns, that produce one type of spore, and staminate, pistillate, male, and female, for plants, such as angiosperms, that produce two types of spores (Subramaniam and Bartlet's (2023) and Meissner’s (2021) suggested uses of “staminate” and “pistillate” overlap but differ.)

As part of this discussion, Pannell (2023) points out that many terms differ in scientific and vernacular meanings, thus this is not a reason to stop using gender in plants. He argues that, when thinking about function, the use of gender in plants is conceptually beneficial, and that alternatives for *male* and *female* would add confusion, not clarity. Furthermore, he discusses how if one thinks of functional gender as a constructed variable describing a sex role (the gamete allocation of a given individual within the context of the gamete allocation of all individuals in the population), that this conceptualization addresses the challenges of applying *female* and *male* to haplodiplontic lifecycles, where the sex-determining life-stage is not always the gamete-producing life-stage. He also states that the context-dependent quantitative use of gender to describe the sex roles of plants is “coherent with some features of these societal trends [about human gender]; we might thus exploit this resonance in opening the botanical world to our students, rather than seeking to avoid it” (p. 2). In Pannell’s (2023) discussions of quantitative gender, as with sex3, organisms only have a gender (or a sex3) while they are in a developmental stage where they are producing gametes. The existence of a null gender (and sex3) is not a shortcoming, rather, it acknowledges that gamete production only occurs at specific developmental stages and may not occur in all individuals.

The current discussion of the use of the term *gender* in botany demonstrates how the use of the same words for different concepts in different taxa has both affordances and challenges and how language is socially constructed in biology. If we were first naming the concept of quantitative gender today, I concur that using another term that does not have the current baggage of *gender* would be useful. However, *quantitative gender* has been in use for nearly 50 years and was coined at a time when societal use of gender was different (Muehlenhard and Peterson 2011). While language change can be vital, and does happen, it requires a large-scale effort within a discipline; furthermore, it also requires knowledge of previous terminology to access older literature (Casper and Warkentin 2026). Because of this history, I concur with Pannell (2023) that continuing to use *gender* in this context has many affordances, and is contextually distanced from the uses of *gender* in humans or a general indiscriminate use of *sex* and *gender* interchangeably. This stance does not mean that I think language should never change. *Quantitative gender* and many other terms in biology with undesired baggage (Schiebinger 2013; Sandford 2023; Subramaniam 2024) demonstrate the need for biologists, collectively, to consider how the challenges and affordances of different language uses harm or help our understanding of and communication about the diversity of life. This consideration needs to involve dialogue by those with wide-ranging disciplinary expertise to navigate the challenges caused by varying meanings and the ill-fitting assumptions, misinterpretations, anthropomorphisms that these can cause.

### Considering changing words: cosexual as an example

Words are often particularly useful if they have contextually consistent meanings that are not easily conveyed by other words. As I have described, many terms have consistent meanings in the technical literature and can be more effectively used if restricted to their technical definitions in broader contexts. In contrast, there are terms, such as cosexual, which do not currently have a coalesced meaning, even within a specific grouping of organisms. While fluidity and evolution of language is also important, a balance of consistency and fluidity is useful for effective communication.

The term cosexual is used in plant and some animal literature to name at least some subsets of organisms that produce both eggs and sperm. Lloyd (1980) explained that he replaced equisexual with cosexual because equisexual problematically implied the equal production of eggs and sperm. Taking Lloyd as a likely origin of cosexual, as per Policansky (1982), Lloyd (1979) also proposed the term *sequential cosexuality* to parallel sequential hermaphroditism in animals. Lloyd (1979, 1980) explicitly situated cosexual as an umbrella term, and used hermaphrodite (paralleling the use of the term synecious) and monoecious to differentiate types of cosexuality.

In the 40+ years since, the term cosexual has been used in a range of ways, including being labeled unnecessary redundant (Cruden and Lloyd 1995). Confusingly, Davies and Singhal (1988) name both sequential cosexuality and cosexuality to be an alternative type of sequential hermaphroditism, “when sequential maturation of testes and ovaries occurs for use in the same breeding [i.e., reproductive] season by outcrossing,” but do not differentiate the two terms (p. 56). Davies and Singhal (1988) and others explicitly contrast sequential cosexuality or cosexuality with protgyny/andry, based on if the sperm and eggs produced are used in the same or different reproductive seasons (Neves and Pires 2002; Cao et al. 2020). In contrast, Pauly et al. (2023) include protogyny/andry in cosexuality. In a different vein, Guest et al. (2012) use cosexual to describe corals where “species that are not simultaneous hermaphrodites have individuals (i.e., polyps and colonies) that are capable of both male and female sex function” (p. 706). And, Cardoso et al. (2018) define cosexual plants as those with only bisexual flowers, (i.e., a synonym for synecious) and exclude some types of sex3 organization that would fit under Lloyd’s cosexual definition, including monoecy, andromonoecy, gynomonoecy, or trimonoecy. And, while Avise (2012) names cosexual as the plant parallel to hermaphrodite in animals, others use cosexual to name specifics of reproductive characteristics in animals, such as leeches and seabasses (Davies and Singhal 1988; Lang and Shain 2009).

This range of uses means that cosexual is neither contextually consistent nor unique in its meaning. When cosexual is used as a synonym of synecious, synecious is clearer. In other cases, such as differentiating changes in gamete production that occur within reproductive seasons from those that occur across breeding/growing seasons, cosexual may fill an unoccupied language niche. In yet other situations, such as using cosexual to replace bisexual to describe flowers that produce both types of gametes, its utility is muddier, since there are already numerous terms to describe this type of flower yet these existing terms can be confusing.

Some have argued that cosexual is a contender for a replacement for the term hermaphrodite, to avoid a word that some consider derogatory, even when not used in relation to humans; tensions about this word can particularly arise in teaching (e.g., Martine 2023; Driessen et al. 2024). Having a broad term to describe the wide range of ways an individual can produce both eggs and sperm sometime during its lifespan is useful, and avoiding potentially contentious language is important when possible. However, any replacement of the term hermaphrodite would require careful consideration and broad input and is beyond the scope of this paper. Deeper conversations with those across a range of biological sub-fields and with those who have intersex and queer identities who are at the center of the potential harms caused by the use of hermaphrodite, are necessary to determine the costs and benefits for broadly replacing hermaphrodite due to its harmful historical use. These conversations may also reveal existing terms, such as synecious, that may already unambiguously fill at least some seeming gaps in more general biological vocabulary.

Thinking from a general language use perspective and drawing from my reading of the literature, cosexual’s most frequent use seems to be in describing simultaneous hermaphroditic organisms, particularly in plants (e.g., Muenchow 1998; Crossman and Charlesworth 2014; Sabatti et al. 2020). This use is also consistent with Lloyd’s (1979, 1980) original definition of the term and with some of the ways it continues to be used. Therefore, even though the uses of cosexual have ranged greatly since its initial coining, using it to mean simultaneous hermaphrodite is the most logical use, leaving *hermaphrodite* for its current broad usage. There is less of a clear pathway for language in relation to sequential hermaphrodites, as *sequential cosexual* would mean “sequential simultaneous hermaphrodite.” For now, the continued use of *sequential hermaphrodite* seems the most logical, as conversations about navigating language in biology are ongoing.

### Moving forward

There is a clear need to support those currently grappling with language in teaching and research, while also addressing inconsistencies, incoherences, and limitations with our current language and how these constrain our ways of conceptualizing sex-related topics in biology. To address current needs, we can use existing language to discuss biological diversity in sex-related topics and teach biology more accurately (e.g., Long et al. 2021; Štrkalj and Pather 2021; Casper et al. 2022; Zemenick et al. 2022; Wright and Delgado 2023; Driessen et al. 2024; Eddy et al., 2026a). Doing this is important because terms can start to lose the utility of their specific meanings when they are (mis)used in overly broad contexts. For example, when the terms dioecious and monoecious are used to mean gonochoric and hermaphroditic, this erases the complexity of the information these terms communicate (Casper and Warkentin 2026). However, despite their clear technical meanings, terms such as monoecious and dioecious do get overgeneralized both within and beyond plants, particularly in more general biology contexts, such as introductory biology textbooks (e.g., Wakim and Grewal 2025). Centering the technical uses of terms that do have clear meanings is important for communication. This focus on better technical language use will also allow us to build foundations for understanding how existing language and conceptions are still insufficient—even biologists specializing in similar areas can come to different conclusions about language use, which can lead to miscommunications and misunderstandings, much of which likely goes undetected (Driessen et al. 2024; Warkentin et al. 2026a).

The need to teach biological complexity and clarify language around sex-related topics has only become starker with the way “biological sex” is being used to create policies and laws for humans that are inconsistent with biology (e.g., Exec. Order No. 14168 2025). Biology is complex, and recent policies and laws rely on definitions that work for discussion of organisms at the population and species level, but not for classifying the developmental variation of every individual. Thus, there is a mismatch between the concepts underlying these terms and how they are being applied for human law and policy. While there is precedent for differences between biological and legal definitions (e.g., the meaning of “vegetable” versus “fruit”), definitions used in law and policy should be informed by biology and acknowledge the simplifications that may be necessary to create biology-informed laws. The example of “leaky” sexes in plants and DSDs in humans demonstrate how some individuals exist at the fuzzy boundaries that exist within sex3 and sex4 (Warkentin et al. 2026a). Universal definitions are not necessarily desirable, but clear definitions within a given context are important; researchers with different questions studying species with different developmental characteristics may need different definitions of similar language. Engaging with language development and clarification is an important part of the dynamic process of science.

While the words we use and their specific meanings will change over time, readers still need to understand earlier literature to engage with prior research. Thus, it is important to define terms and engage with the complex histories of language in biology. There are calls within biology, particularly in the plant literature, to change our language and thereby our conceptualization of sex and structures related to it (e.g., Meissner 2021; Diggle 2023; Oberle and Fairchild 2023; Pannell 2023; Subramaniam and Bartlett 2023). This paper does not refute these calls. Rather, it clarifies language we can use better now, while we take time for the deeper conversations that are necessary to better address the fuzzy boundaries and conceptual unclarity around sex, gender, and reproduction.

## Data Availability

Data sharing is not applicable to this article, as no new data were created or analyzed in this study.

## Footnotes

1 There is one reported case of a domestic rabbit that was identified as male due to its external genitalia and successful siring of offspring with female rabbits; however, it later produced one viable self-fertilized litter (Frankenhuis et al. 1990). It was autopsied during its second pregnancy, which revealed fetuses developing typically and both ovaries and testes. At the time the ovaries were functional but the testes were not; pregnancy hormones would likely cause the testes to become infertile. It’s sex could not be determined chromosomally, due to unsatisfactory chromosome preparations. Across its litters it bore female and male offspring in equal proportion, indicating that it was likely an XX/XY mosaic. The authors were unsure how self-fertilization occurred, however they hypothesized that semen was ejaculated from the penis-like clitoris, an opening that was part of the vaginal wall.

## Notes

From the symposium “Sex across origins: Questioning animal-centric assumptions and developing integrative frameworks” presented at the annual meeting of the Society for Integrative and Comparative Biology, January 3–7th, 2026.
